# New bis(azobenzocrown)s with dodecylmethylmalonyl linkers as ionophores for sodium selective potentiometric sensors

**DOI:** 10.1007/s10847-016-0661-9

**Published:** 2016-09-29

**Authors:** Elżbieta Luboch, Maciej Jeszke, Mirosław Szarmach, Natalia Łukasik

**Affiliations:** Department of Chemistry and Technology of Functional Materials, Faculty of Chemistry, Gdańsk University of Technology, Narutowicza 11/12, 80-233 Gdańsk, Poland

**Keywords:** Bis(azobenzocrown)s, Synthesis, Sodium ionophores, Ion-selective electrodes, Solid contact electrodes

## Abstract

Novel biscrowns **1** and **2** were synthesized from 13-membered azobenzocrown ethers containing bromoalkylenoxy chains in *para* position relative to the azo group. The synthesized diester molecules are dodecylmethylmalonic acid derivatives differing by the linker length. The synthesized compounds have the potential of being used as sodium ionophores in ion-selective electrodes. They were characterized and used as ionophores in classic and miniature, *solid contact* (screen-printed and glassy carbon) membrane ion-selective electrodes. Compound **3**, a similar monoester derivative of 13-membered azobenzocrown, was synthesized and used in membrane electrodes for comparison. Lipophilicity of new ionophores was determined by TLC. Lipophilicity of bis(azobenzocrown)s was found to be within the range of logP_TLC_ = 12–13. It was observed that the particularly important selectivity coefficients log*K*_*Na,K*_ determined for new electrodes, being log*K*_Na,K_ = −2.5 and −2.6 (SSM, 0.1 M), are better than those of the electrodes featuring seven out of the nine commercially available sodium ionophores. It was concluded that the ionophore **1** creates, in acetone, with sodium iodide, complex of 1:1 stoichiometry (sandwich complex) with stability constant (log*K*) ca. 3.0.

## Introduction

Molecular receptors based on macrocyclic compounds are established as complexation agents for metal cations. An interesting group of alkali metal complexation compounds consists the crown ethers incorporating azobenzene moieties as part of the macrocycle, i.e. azobenzocrown ethers. Numerous macrocyclic compounds with inherent 2,2′-azobenzene moieties were synthesized and studied exhaustively [1–4]. Broad possibilities for functionalization of azobenzocrowns result in their potential applications in a variety of fields, e.g. lipophilic crowns may be successfully used as ionophores, both in classic and miniature *solid contact* ion-selective membrane electrodes (ISEs) [2–4].

Azobenzocrowns with peripheral hydroxyl groups are compounds with chromoionophoric and fluoroionophoric properties [5–7]. At the same time, they are convenient substrates for further modifications of azobenzocrowns and allow introduction of various peripheral functional groups including derivatives featuring reactive bromine atoms within the side chains [8]. Chromoionophoric properties are also observed e.g. for azobenzocrowns featuring amine substituents within the benzene ring and *pull*–*push* type azobenzocrowns featuring nitro and dimethylamine groups within the benzene rings [2, 9].

In our earlier studies we have found that ISEs with 13-membered azobenzocrown ionophores in membranes are sodium selective. We obtained and used in the membrane ion-selective electrodes a number of 13-membered azobenzocrowns with modifications within the benzene rings, including crowns with: hydrocarbon moieties, hydrocarbon moieties linked by an ether group, compounds with ester or amide groups within the side chains or bis(azobenzocrown)s with two azobenzocrowns connected via an α,ω-dioxaalkane [2–4].

Most typically, ISEs based on crown ethers ionophores are selective towards ions (including sodium cations) that form sandwich-type complexes with crown ethers [10–12] (the exception being crown ethers that contain blocking subunits disallowing formation of complexes with cations larger than the macrocycle gap, e.g. [13]). It was previously reported that the 13-membered azobenzocrowns form crystalline sandwich-type complexes (2:1, crown:cation) with sodium ions, with the complexed cations being well-separated from the anions [12]. Macrocycles of this ring size have complexing properties similar to those of 12-crown-4 [14]. Conditions for the formation of sandwich-type complexes may be favored when two macrocycles are appropriately linked to form bis(crown ether)s. Due to cooperation of the neighboring crown units, biscrown derivatives tend to form stronger complexes with the particular ions than their respective monocrowns [15]. The linker binding the two crown units has a significant effect on the formation of sandwich complexes by biscrown ethers [16]. Both the length and the flexibility of the linker are of importance e.g. [17]. Intramolecular sandwich complexes cannot be formed in case of rigid molecules where the crowns lie either too far apart or too close to each other. However, intermolecular complexes may be formed instead. In particular cases, a 2:2 (biscrown:ion) stoichiometry is possible, with two cations being shared by two ligand molecules [18, 19]. Such is the case, for example, in bis(azobenzocrown) with a short, dioxyethylene linker that forms an intermolecular sandwich-type complex of 2:2 stoichiometry with sodium ions [3].

Recently, much attention was focused on the use of ISEs for clinical analysis [20]. The sensors, forming part of commercial analyzers, are applied for the measurement of ions such as sodium, potassium, calcium, magnesium, lithium, ammonium, hydrogen, chloride, and bicarbonate. Ionophores for use in ISEs applied for determination of all these ions are available commercially. One to nine ionophores are offered for each ion [21]. However, the literature contains reports regarding much higher numbers of ionophores, sometimes including some that seem more interesting but have not been commercialized for various reasons eg. [22]. For example, the sensational ISEs featuring calix[4]crown-4, selective towards sodium ions, absorb proteins which prevents them from being used in measurements carried out in blood or cell cultures [23]. As observed in an example of azobenzocrowns, different from those reported herein, azobenzocrowns with no structural similarity to natural compounds have no affinity towards protein molecules [24].

High selectivities, e.g. for sodium-selective electrodes, particularly in the case of calix[4]arene ionophores application, are obtained when the ISE did not have any contact with the primary ion before the measurement. However, usually the again used sodium-selective electrode shows even two orders of magnitude worse sodium/potassium selectivity coefficient [13, 25].

We are interested in the search for new, interesting ionophores for electrodes to be used for clinical analytics, including sodium electrodes. Although commercial sodium ionophores are quite numerous when compared to other ionophores, not many highly lipophilic compounds characterized by very good sodium/potassium selectivity are available [21]. Lack of interference with calcium and magnesium ions is also important from the clinical analytics point of view.

Table 1 presents the properties of sodium-selective membrane electrodes suitable for clinical analytics in terms of both selectivity and lipophilicity [26].

**Table 1:** ISE selectivities required for determination of sodium ions in clinical analytics and minimum recommended lipophilicity of the ionophore [26]

Required ISE selectivity in plasma and ionophore lipophilicity	Required ISE selectivity in urine and ionophore lipophilicity
log*K*_Na/K_ < −0,6 log*K*_Na/Ca_ < −1,3 log*K*_Na/Mg_ < −1,2 log*K*_Na,H_ < 4.4 logP_TLC_ > 8.4	log*K*_Na/K_ < −2,1 log*K*_Na/Ca_ < −0,6 log*K*_Na/Mg_ < −0,6 log*K*_Na,H_ < 1.4 logP_TLC_ > 2.3

Among the nine commercially available sodium ionophores (Fluka, Sigma-Aldrich) can be distinguished: podands (diamides and triamides), crown ethers (including biscrowns), an ester derivative of calixarene and monensin methyl ester [21].

**Fig. 1:**
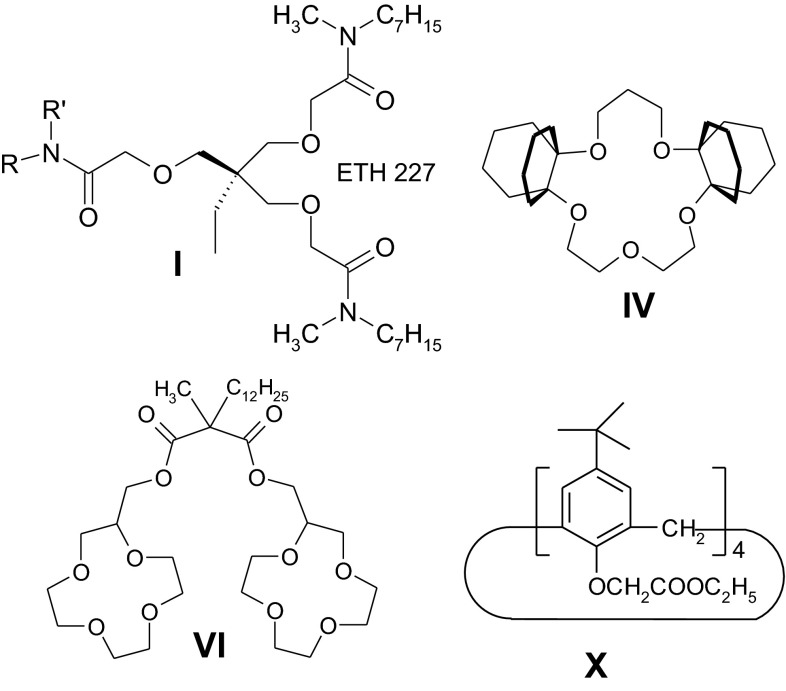
Commercial sodium ionophores: **I**, **IV**, **VI** and **X** (ionophore symbols by Fluka)

In Fig. 1 the most important sodium ionophores are shown representing four groups of compounds: acyclic triamides, crown ethers, biscrowns, and derivatives of calix[4]arene.

In the case of amide-based podands, e.g. sodium ionophore **I** different selectivity log*K*_Na,K_ ranging from −0.2 was obtained depending on ISE type and measurement conditions [21]. A satisfactory value of selectivity coefficient for potassium can be also gained, e.g. **I** (SSM 0.1 M, *o*-NPOE, TPB^−^): log *K*_Na,K_ = −2.3, however not good enough discrimination of lithium and calcium ions may be a problem (log *K*_Na,Li_ = 0.4; log *K*_Na,Ca_ = 0.2) [27]. By changing a plasticizer the improvement of calcium selectivity can be achieved (**I**, BBPA (SSM): log *K*_Na,K_ = −2.0; log *K*_Na,Li_ = 0.5; log *K*_Na,Ca_ = −1.5) [28].

High discrimination of potassium ions can be obtained by introduction to 16-crown-5 ring of two bulky decaline units to prevent the formation of 2:1 complexes by the crown with potassium ions (ionophore **IV**, BBPA + TEHP (FIM): log *K*_Na,K_ = −3.0; log *K*_Na,Li_ = −3.1; log *K*_Na,Ca_ = −4.0) [13]. A disadvantage of this ionophore is its high price.

Relatively high discrimination of potassium and lithium ions can be achieved by using of biscrown ionophores with two 12-crown-4 units, that create sandwich complexes with sodium ions (e.g. ionophore **VI** (MSM, *o*-NPOE): log *K*_Na,K_ = −2.0; log *K*_Na,Li_ = −3.0; log *K*_Na,Ca_ = −4.0; log *K*_Na,Mg_ = −4.0) [14]. Sodium ionophores from the biscrown group usually discriminate calcium and magnesium ions strongly.

The unquestionable leader of the ionophore sodium is currently ionophore **X**—*p*-*tert*-butylcalix[4]arene-tetraacetic acid tetraethyl ester, one of many derivatives of calix[4]arene (ester- and amide-based), selective for sodium ions which have been described in literature eg. [22]. The ionophore **X**, much cheaper than ionophore **IV**, characterized in a little bit poorer selectivity for potassium ions (e.g. **X** (SSM, *o*-NPOE): log *K*_Na,K_ = −2.7; log *K*_Na,Li_ = −3.4 [29]) is commonly used in most studies of the potentiometric sodium ions determination.

Recent researches, where among the others sodium-selective ionophores are used, are focused more on new technical solutions and new areas of sensor systems application than on search for novel, selective ionophores, e.g. [30–34].

**Scheme 1:**
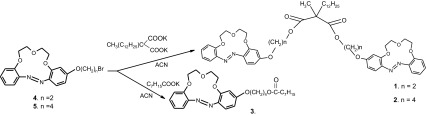
Method of synthesis and structures of the new ionophores tested in sodium selective membrane electrodes

Now, new diester bis(azobenzocrown)s, derivatives of dodecylmethylmalonic acid, were synthesized, being somewhat analogous to the known, commercially available sodium ionophore **VI** [35]. The new compounds **1** and **2** (Scheme 1), as well as the new monoester derivative **3** acting as the comparative ionophore, were used in classic membrane ion-selective electrodes and miniature electrodes of the *solid contact* type: glassy carbon and graphite screen-printed. As the ionophores are photoactive compounds having ability to isomerization, the influence of light in UV range on obtained electrodes properties as well as the presence of sodium ions on isomerization of tested bis(azobenzocrown)s was examined. The stoichiometry of complex of bis(azobenzocrown) **1** with sodium ions was determined.

## Experimental

### General

Materials (mainly from Sigma-Aldrich) and solvents (mainly from POCH, Poland) for synthesis were of analytical reagent grade and used without further purification. Silica gel 60 (0.063–0.200 mm) (Merck) was used for column chromatography. TLC: silica gel 60F_254_-coated aluminum sheets were purchased from Merck. TLC determination of lipophilicity was conducted using RP-18 F254S, 0.25 mm plates (Merck) and bis(2-ethylhexyl)sebacate (DOS), bis(2-ethylhexyl)phthalate (DOP), bis(1-butylpentyl)adipate (BBPA) and *o*-nitrophenyl octyl ether (*o*-NPOE) (Sigma-Aldrich) as plasticizer references. Tetrahydrofuran (THF) for membrane preparation was freshly distilled over LiAlH_4_. For the preparation of polymeric ion-selective membranes, poly(vinyl chloride) (PVC, high molecular weight), potassium tetrakis(4-chlorophenyl)borate (KT*p*ClPB), poly(sodium 4-styrenesulfonate) (NaPSS), ethylenedioxythiophene (EDOT), poly(3,4-ethylenedioxythiophene)/poly(styrenesulfonate) blend (PEDOT/PSS, used as 1.3 % (w/w) dispersion in water (conductive grade)), *p*-*tert*-butylcalix[4]arene-tetraacetic acid tetraethyl ester (Sodium Ionophore **X**) and bis[(12-crown-4)methyl]dodecylmethylmalonate (Sodium Ionophore **VI**) were used as received from Sigma-Aldrich.

The screen-printed graphite electrodes were prepared in the Institute of Electronic Materials Technology, Warsaw (plates of 18–15 mm with six electrodes, opening areas of ca. 1 mm^2^, substrate—polyester film, 125 μm thick, type CT-5 (Autostat), dielectric paste type 5018 (Du Pont, UV-cured). Carbon nanotubes (Thin MWCNT 95 %) were obtained from Nanocyl S.A., Belgium.

Glassy carbon electrodes (glassy carbon disk of 1.8 mm in diameter in poly(ether ether ketone) casing were obtained from Mineral^®^ (Warsaw, Poland).

The aqueous solutions were prepared with deionized water of conductivity below 0.1 μS/cm, obtained using a Hydro-Lab-PL reverse osmosis (RO) station.

^1^H NMR spectra were recorded on a Varian instruments at 500 MHz. Mass spectra were recorded on a SYNAPT G2-S HDMS (Waters) (ESI) spectrometer. FTIR spectra were recorded using a Mattson Genesis II instrument. UV–Vis spectra were recorded using a UNICAM UV 300 apparatus. All EMF was measured with 16–channel potentiometer (Lawson Labs. Inc., USA) connected to a computer with EMF Suite data logging software version 2.0. pH was monitored using a pH-meter CX-505 and electrode EPP-1 (ELMETRON).

### Synthesis

The ionophores were prepared as shown in the Scheme 1.

Potassium dodecylmethylmalonate (54.3 mg, 0.15 mmol), the bromo derivative **4** or **5** [8] (122 or 130 mg, 0.30 mmol) and a catalytic amount of 18-crown-6 in dry acetonitrile (5 ml) were heated at 75 °C for 24 h. Then, the solvent was evaporated under reduced pressure and the residue was separated by column chromatography on silica gel. Compounds **1** and **2** may theoretically occur as three stereoisomers, two of which were mainly present in solutions containing the product. One of the isomers was eluted from the column with a mixture of chloroform:acetone 5:1 and the other with a mixture of chloroform:methanol 5:1. After comparison of chromatograms of individual fractions (TLC) (after about 1 h), fractions containing stereoisomers of individual compounds were combined and solvents were evaporated under reduced pressure. Oily residues were crystallized from ethyl acetate:hexane mixture. Compound **1** (red–orange solid) was obtained in a 38 % yield, mp 84–87 °C. Compound **2** (red–orange solid) was obtained in a 43 % yield, mp 57–58 °C (mainly *trans*–*trans* isomer).

Compound **1** was treated with an equimolar amount of sodium iodide dissolved in methanol. The resulting mixture was evaporated under reduced pressure and, after several hours, subjected to ^1^H NMR analysis in d-acetone.

Compound **1**. TLC: R_f_ = 0.20, 0.33 and 0.51 for *trans*–*trans*, *trans*–*cis* and *cis*–*cis* isomers respectively (dichloromethane:acetone, 4:1, v/v); R_f_ = 0.31, 0.76 and 0.81 for *trans*–*trans*, *trans*–*cis* and *cis*–*cis* isomers respectively (chloroform:methanol, 10:1, v/v). ^1^H NMR (d-acetone), mixture of isomers: 0.84–0.90 (3H, m); 1.20–1.32 (20H, m); 1.38–1.44 (3H, m); 1.80–1.92 (2H, m); 3.60 (~1.3H, s); 3.86–4.60 (~22.7H, m); 6.30–7.80 (14H, m). FT-IR (film): C = O 1733 cm^−1^. UV–Vis (acetone): λ(ε) = 361 nm (2.3 × 10^4^); 437 nm (2.7 × 10^3^). HRMS (ESI): [M+Na]^+^ 961.4570 calculated for C_52_H_66_N_4_O_12_Na 961.4575.

Compound **1**·NaI. ^1^H NMR (d-acetone), signals for *trans*–*trans* isomer (ca. 90 % of content): 0.85–0.90 (3H, m); 1.22–1.39 (20H, m); 1.46 (3H, s); 1.91–1.94 (2H, m); 3.94–4.05 (8H, m); 4.21–4.40 (12H, m); 4.51 (4H, t, *J* = 5 Hz); 6.67–6.72 (4H, m); 7.01 (2H, d, *J* = 8 Hz); 7.07 (2H, t, *J* = 8 Hz); 7.23 (2H, t, *J* = 7 Hz); 7.34 (2H, d, *J* = 8 Hz); 7.38 (2H, d, *J* = 9 Hz).

Compound **2**. TLC: R_f_ = 0.04, 0.48 and 0.57 for *trans*–*trans*, *trans*–*cis* and *cis*–*cis* isomers respectively (dichloromethane:acetone, 4:1, v/v); R_f_ = 0.18, 0.82 and 0.87 for *trans*–*trans*, *trans*–*cis* and *cis*–*cis* isomers respectively (chloroform:methanol, 10:1, v/v). ^1^H NMR (d-acetone), signals for *trans*–*trans* isomer: 0.84–0.88 (3H, m); 1.27–1.39 (20H, m); 1.41 (3H, s); 1.87–1.92 (10H, m); 3.86–3.94 (8H, m), 4.12 (4H, t, *J* = 6 Hz); 4.21–4.29 (12H, m); 6.71 (2H, d, *J* = 1 Hz); 6.74 (2H, d, *J* = 2 Hz); 7.13–7.18 (4H, m); 7.35 (2H, t, *J* = 7 Hz); 7.65 (2H, d, *J* = 8 Hz); 7.73 (2H, d, *J* = 9 Hz). FT-IR (film): C = O 1730 cm^−1^. HRMS (ESI): [M+Na]^+^ 1017.5187 calculated for C_56_H_74_N_4_O_12_Na 1017.5201.

Compound **3** was synthesized in an analogous manner using potassium octoate (54.6 mg, 0.30 mmol). The product was eluted from the column with methylene chloride:acetone 5:1 and then crystallized in mass. Yield 64 %, mp 47–49 °C (red solid). TLC: R_f_ = 0.50 and 0.77 for *trans* and *cis* isomers respectively (dichloromethane:acetone, 4:1, v/v); R_f_ = 0.76 and 0.85 for *trans* and *cis* isomers respectively (chloroform:methanol, 10:1, v/v).

^1^H NMR (d-acetone), mixture of *trans* and *cis* isomers (3:2): 0.88 (3H, t, *J* = 7 Hz); 1.24–1.36 (8H, m); 1.55–1.64 (2H, m); 1.75–1.94 (4H, m); 2.27–2.34 (2H, m); 3.87–4.32 (12H, m); 6.30 (0.4H, dd, *J*_1_ = 9 Hz, *J*_2_ = 2 Hz); 6.51 (0.4 H, d, *J* = 9 Hz); 6.58 (0.4H, d, *J* = 2 Hz); 6.75–6.78 (1.2H, m); 6.83–6.88 (0.8H, m); 6.94 (0.4H, d, *J* = 8 Hz); 7.10 (0.4H, dd, *J*_1_ = 8 Hz, *J*_2_ = 2 Hz); 7.16 (0.6H, t, *J* = 8 Hz); 7.20 (0.6H, d, *J* = 8 Hz); 7.37 (0.6H, t, *J* = 8 Hz); 7.69 (0.6H, d, *J* = 8 Hz); 7.78 (0.6H, d, *J* = 10 Hz). FT-IR (film): C = O 1732 cm^−1^. HRMS (ESI): [M+H]^+^ 499.2798, calculated for C_28_H_39_N_2_O_6_ 499.2808.

### Ion selective electrodes membrane preparations and EMF measurements

Membranes containing the ionophores **1**, **2** or **3** (6 mg, 3.8 % (w/w)), PVC (50 mg, 31.8 wt%), *o*-NPOE (100 mg, 63.7 % (w/w)) and KT*p*ClPB (1.1 mg, 0.7 % (w/w)) were prepared for classical electrodes (**c**). All components were dissolved in 1.2 mL of freshly distilled THF and transferred to a glass ring (15 mm i.d.). The solvent was allowed to evaporate overnight at ambient temperature. From obtained membrane discs (7 mm dia.) were cut out and mounted onto Philips electrode bodies (W. Moller AG, Zurich). Next, the membrane electrodes were soaked in 10^−2^ M NaCl overnight prior to use. Philips silver chloride electrode (W. Moller AG, Zurich) was used as the reference electrode in the measurements. Potentials were measured using the following galvanic cell: Ag | AgCl(s) |KCl (1 M) | CH_3_COOLi (1 M) | sample solution | ion-selective membrane | NaCl (10^−2^ M) | AgCl(s) | Ag.

For screen-printed electrodes (**s**-**p**) the membranes of analogous composition as for classical electrodes were prepared. Carbon nanotubes were optionally added to membrane to be deposited on graphite screen-printed electrodes (**s**-**p**,**n**). Carbon nanotubes (0.05 mg) in 1.2 mL THF were sonicated for 15 min. Next, the remaining components were added in quantities typical for classic electrodes. The resulting cocktail (1 μL) was dosed onto graphite screen-printed electrodes (membrane surface area of ca. 1.5 mm^2^). After 5 min, another, identical portion of the cocktail was applied. The electrodes were left for solvent evaporation over at least 6 h at ambient temperature. Next, the electrodes were soaked in 10^−3^ M NaCl for 10 h prior to use. The same reference electrode was used as in the case of classic electrodes.

The conductive polymer layer (PEDOT/PSS, 5 μL) was transferred onto glassy carbon electrodes **gc** (application area: disc 1.8 mm in dia.). The electrodes were left for 24 h at room temperature before the deposition of the membrane (15 μL) onto an area of ca. 4 mm in diameter. After 5 min, another, identical portion of the cocktail was applied. Electrodes were left overnight at ambient temperature. Later, the membrane electrodes were soaked in 10^−3^ M NaCl overnight. The composition of membranes was analogous as for classical electrodes, but here also DOS instead of *o*-NPOE was used (in the same quantity).

Composition of the membranes containing the reference calixarene tetraester (Sodium Ionophore **X**, SI-**X**) was as follows: ionophore (2 mg, 1.3 % (w/w)), PVC (49.5 mg, 32.8 % (w/w)), *o*-NPOE (99.2 mg, 65.7 % (w/w)), and KTpClPB (0.3 mg, 0.2 % (w/w)).

Composition of the membranes containing the reference bis[(12-crown-4)methyl]dodecylmethylmalonate (Sodium Ionophore **VI**, SI-**VI**) was as follows: ionophore (5.2 mg, 6.5 % (w/w)), PVC (21.44 mg, 26.8 % (w/w)), *o*-NPOE (53.36 mg, 66.7 % (w/w)).

Alternatively, for one, comparative series of electrodes (**gc′**) with ionophore **1** the conductive polymer was introduced by electropolymerization. Films consisting electroactive polymer were prepared by direct electropolymerization on the glassy carbon electrodes from the suspension (in electrolyte) containing the monomer (0.0066 M EDOT) and poly(sodium 4-styrenesulfonate) (0.05 M NaPSS). Electrodeposition of PEDOT/PSS was carried out potentiostatically (0.95 V vs. Ag/AgCl in 0.1 M KCl, t = 200 s, Q = 6,6 mC), under argon atmosphere, by the potentiostat–galvanostat AutoLab PGStat302 N system under GPES software control.

Characteristics of the electrodes were examined by measuring the cell EMF for aqueous solutions of given salts (NaCl, KCl, LiNO_3_, NH_4_Cl, CaCl_2_, MgCl_2_) and HCl (for chosen electrodes) over the concentration range of 10^−7^–10^−1^ or 1 M. All measurements were carried out at room temperature (ca. 20 °C). The selectivity coefficients (log *K*) were determined by the Separate Solution Method (SSM) [36] according to the following formula using 0.1 M solutions of the salts:

$$ \log K_{IJ}^{pot} = \frac{{\left( {E_{J} - E_{I} } \right)}}{{S_{I} }} + \log \left( {a_{I} } \right) - \frac{{z_{I} }}{{z_{J} }}\log \left( {a_{J} } \right) $$

*E*_*J*_—interfering ion potential; *E*_*I*_—main ion potential; *S*_*I*_—main ion characteristics slope; *a*_*I*_ *, z*_*i*_—activity and charge of main ion; *a*_*J*_ *, z*_*j*_—activity and charge of interfering ion.

The correlation between the potential and pH was tested for constant NaCl concentrations (10^−1^, 10^−2^ and 10^−4^ M) and variable concentrations of HCl or LiOH.

In order to determine the levels of sodium in the real-life sample, a solution comprising KCl (4.2 mM), CaCl_2_ (1.1 mM), and MgCl_2_ (0.6 mM) was prepared, further referred to as “artificial plasma”. Artificial plasma was used as background for characterization of the electrodes used for determination of sodium levels in real-life plasma samples. The activity of sodium ions in plasma was determined by direct measurement method.

### Examination of UV light effect on response of electrode containing biscrown ionophore **1**

The properties of **gc′** type electrodes with compound **1** as an ionophore was tested. In next step the membranes of those electrodes were irradiated for 1 h with a lamp emitting light of 365 nm (power of 9 W). Immediately after irradiation another series of measurements was performed. After 1 h and next after 1 day of electrodes conditioning the measurements were repeated.

Independently, the acetone solution of compound **1** was irradiated (UV, 365 nm, 9 W). Immediately after the irradiation only a small amount of the *trans*–*trans* isomer was observed (TLC). Its content was estimated at approximately 5 %. ^1^H NMR spectrum was also registered directly after irradiation of compound **1** in d-acetone. The content of particular isomers was estimated as: *cis*–*cis* isomer ca. 65 %, *trans*–*cis* isomer ca. 30 %, *trans*–*trans* isomer ca. 5 % (Fig. 4).

### Determination of lipophilicity of the ionophores **1**, **2** and **3**

Lipophilicity of new ionophores was determined by TLC [37] using reverse-phase chromatographic plates and ethanol–water 10:1 as the mobile phase. DOP, DOS, *o*-NPOE, and BBPA were used as the reference standards. Lipophilicity values calculated for the compounds **1**, **2**, and **3** were log P_TLC_ = 12.0 ± 0.3, 13.1 ± 0.2, and 7.0 ± 0.5, respectively.

### Determination of the stability constant values and stoichiometry of complex of biscrown **1** with sodium iodide

UV–Vis measurements were recorded in acetone using quartz cuvettes (0.2 cm). The spectral changes of ligand solution of defined concentration (c_L_ = 1.12 × 10^−4^ M, 0.4 mL), were recorded upon stepwise addition of 40 µL of the salt solution (titration step) directly into the measurement cell (concentration of stock solution of the salt was 8.17 × 10^−3^ M). Spectrophotometric titration was ended at concentration ratio c_NaI_/c_L_ = 43.82 (after addition of 240 µL of stock solution of salt into measurement cell). On the basis of experimental data the stability constant values and stoichiometry of species were determined using the OPIUM software [38].

## Results and discussion

### Synthesis

The synthetic approach to novel ionophores is shown in Scheme 1.

Suitable bromo derivatives [8] were subjected to reaction with potassium salts of acids. Reactions were carried out in acetonitrile (ACN) at 75 °C. An analogous reaction with silver salt and a synthetic method featuring dodecylmethylmalonic acid dichloride and hydroxyl derivatives of alkoxyazobenzocrowns were also tested, but the best results were achieved using the method outlined in Scheme 1. The yield of the synthesis and isolation of biscrowns was about 40 %.

**Fig. 2:**
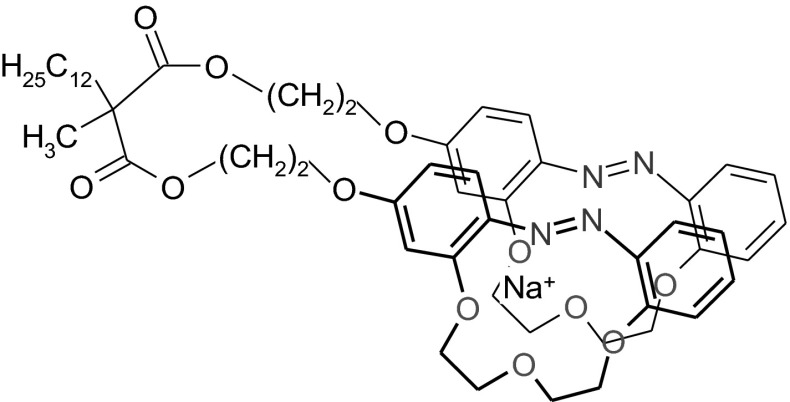
Schematically, the probable structure of complex of biscrown **1** with sodium ion

Equilibria between the two geometric isomers of azobenzocrown **3**, i.e. *trans* and *cis* isomers and between the three isomers, i.e. the *trans*–*trans, trans*–*cis* and *cis*–*cis* isomers of bis(azobenzocrown)s **1** and **2**, are observed in the solutions. In acetone solution of the ionophore **3** a typical for derivatives of 13-membered azobenzocrown ratio of *trans* to *cis* isomers being 6:4 (^1^H NMR) was observed. In solutions of biscrowns **1** and **2** the *trans*–*trans* and *trans*–*cis* isomers were dominant representing in sum approximately 90 % of total amount of the compounds. For instance, for acetone solution of biscrown **1** the content of *trans*–*trans*, *trans*–*cis* and *cis*–*cis* stereoisomers was estimated at 5.5:3.5:1 respectively (^1^H NMR). Malonate bis(azobenzocrown)s are characterized by large differences in chromatographic properties (TLC,) of *trans*–*trans* and *trans*–*cis* isomers. The difference is particularly large for compound **2**. On the basis of our many years, extensive experience with this type of compounds e.g. [4] we may conclude that this may indicate distinctly different complexation properties of these isomers in favor of the *trans*–*trans* isomer. The *trans*–*trans* isomer should be capable of forming an intramolecular sandwich-type complex (Fig. 2) while the *trans*–*cis* isomer should rather form intermolecular complexes due to the poor complexation capabilities of the *cis* isomers of azobenzocrowns. This finds confirmation in our previous results as well in researches published by other authors [1, 12, 39].

### The interaction of the ionophore **1** with sodium ions in acetone solution

**Fig. 3:**
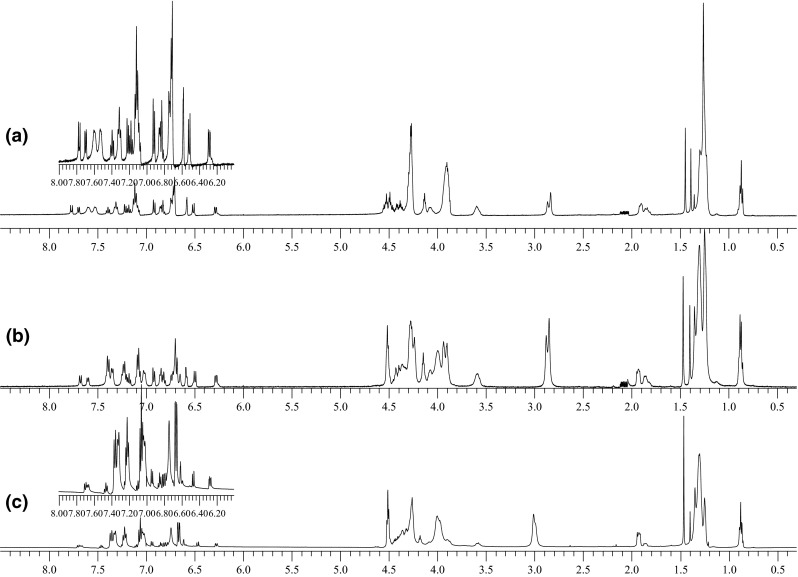
^1^H NMR spectra of compound **1** in d-acetone: **a** without salt, **b** spectrum registered directly after addition of equimolar amount of NaI, **c** spectrum in the presence of the salt registered after several hours. (Acetone peaks removed for clarity)

Comparison of ^1^H NMR spectra of compound **1** (d-acetone) and the spectra of the same compound in the presence of sodium iodide revealed that the presence of sodium ions shifts the equilibrium towards the predominance of the *trans*–*trans* isomer (Fig. 3). Obtained results suggest, that formation of complex with sodium ions by *trans*–*trans* isomer is strongly preferred.

In ^1^H NMR spectrum of bisazobenzocrown **1** (Fig. 3) three ranges can be distinguished:

1. The first one is range from 0.8 to 2.0 ppm, which can be assigned to protons of aliphatic hydrocarbon groups not adjacent to oxygen atoms. In this range changes of singlets intensity at 1.45, 1.39 and 1.36 ppm corresponding to *trans*–*trans*, *trans*–*cis*, and *cis*–*cis* isomers respectively, can be tracked. Those signals practically do not change their position in the presence of the sodium salt. Also, in the range of 1.80–1.95 ppm, where signal of CH_2_ dodecyl fragment is present changes of signal intensity and position depending on isomeric composition of biscrown can be seen. Shifts of this signal due to complexation is also not observed. For *trans*–*trans* isomer shift of this signal is 1.90 ppm.
2. The second range is 3.5–4.6 ppm corresponding to protons bound with ether carbon atoms including both protons of macrocycle and protons ethylene moieties from ester part of the ligand. It is a complex system of partly overlapping signals. From this range it can be concluded about the participation of macrocycles with *cis*-azobenzene moiety, on the basis of signal integration at 3.6 ppm corresponding to protons at “central” carbon atoms of macrocycle. One of the most important changes in this part of ^1^H NMR spectra of the free ligand and its complex is a shift of signal (s) of four protons: ArOCH_2_–two from each macrocycle from 4.3 to 4.5 ppm as a consequence of sodium ions complexation by *trans*–*trans* isomer.
3. The third range is 6.2–7.8 ppm. A complicated system of multiplets corresponding to seven signals of *trans*–*trans* isomer, fourteen signals of *trans*–*cis* isomer, and seven signals of *cis*–*cis* isomer is here observed (in Fig. 3 they are hardly visible). The most readable is the c) spectrum (Fig. 3) for complex of *trans*–*trans* isomer of the biscrown with sodium iodide, where the *trans*–*trans* isomer is dominant (~90 %). However, a clear proton shielding with comparison to free ligand spectrum is here observed (Fig. 3a). The most characteristic is shift of protons from *ortho* position to azo group (from 7.5–7.7 to 7.3–7.4) indicative of the reduced electron-donor properties of azo group, taking probably part in sodium ions complexation. Similar changes were observed in the spectra of the complex of the parent, 13-membered azobenzocrown acquired in CDCl_3_ [12].

**Fig. 4:**
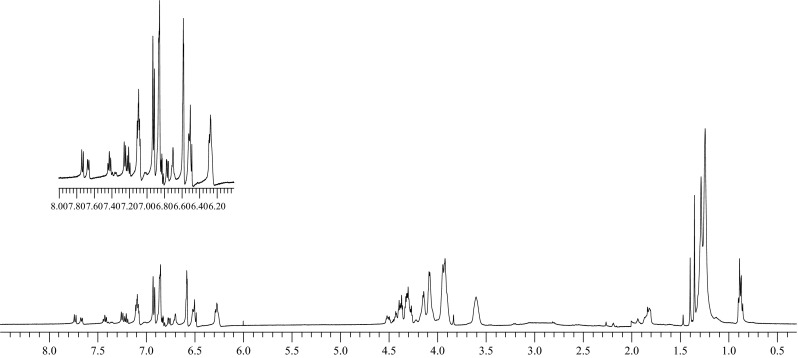
^1^H NMR spectrum of compound **1** in d-acetone after irradiation with UV light (365 nm) for 1 h. (Acetone and water peaks removed for clarity)

Signals characteristic for *cis*–*cis* isomer are the most visible in ^1^H NMR spectrum of biscrown irradiated with UV light (Fig. 4), where content of *cis*–*cis* isomer was estimated as 65 %.

**Fig. 5:**
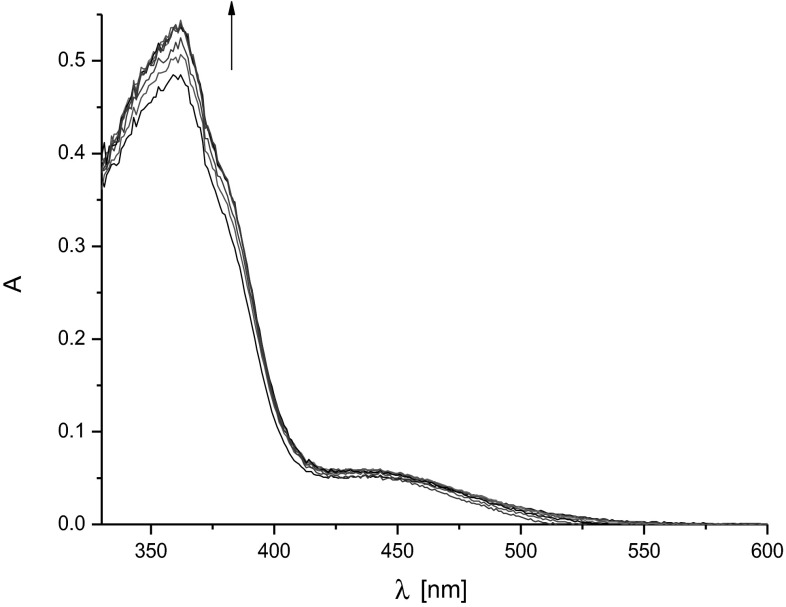
Changes in UV–Vis spectrum of ligand **1** solution (c_L_ = 1.12 × 10^−4^ M) in the presence of sodium iodide (c_NaI_: 0–3.06 × 10^−3^ M) in acetone

Althought, changes in ^1^H NMR spectra in the presence of salt are evident, chemical shifts of particular signals connected only with complexation process are not large enought for reliable determination of stoichiometry of formed complexes. This is why, interactions of compound **1** with sodium iodide were also tested using UV–Vis spectrophotometry. The presence of salt in ligand solution in acetone causes hyperchromic shift of ligand spectrum (Fig. 5). The system equilibrates at large molar excess of salt (c_NaI_/c_L_ = 10). On the basis of analysis of experimental data the most probable binding model for **1**-NaI system is 1:1 stoichiometry (intramolecular sandwich complex, Fig. 2). The stability constant estimated for created species using OPIUM software is log*K* ~ 3.0.

In the case of small macrocycles, and of lack of polarizing or ionizing moieties in azobenzocrown structure spectacular changes in UV–Vis spectra of azobenzocrown and their complexes of sandwich type are not seen (e.g. [3]).

### Membrane ion-selective electrodes

We present the results of research for potentiometric ion selective membrane electrodes with three new sodium ionophores **1**, **2** and **3**. The use of these compounds, being capable of isomerization, as ionophores in ISEs, does not pose problems. Azobenzocrown compounds have been used as ionophores for two decades. Equilibrium is established between the isomers within the liquid membrane and thus the electrodes may operate correctly even when pure *cis* isomer is introduced. The *trans* isomer is predominant in the equilibrium in solution for 13-membered or larger macrocyclic azocompounds, whereas in the case of smaller azobenzocrowns, e.g. 10-membered, the *cis* isomer is characterized by slightly higher stability than the *trans* isomer [40]. Upon the contact of the membrane with a solution containing the preferred ion, the equilibrium should be even more shifted towards the *trans* isomer (or *trans*–*trans* in case of bis(azobenzocrown)s). In addition, the access of light may be reduced during the measurements. The effect of light on the results of potentiometric studies was preliminary studied by means of measurements conducted in darkness. No significant differences in electrode characteristics were observed. Only after irradiation of electrode membranes with a lamp emitting UV light with power of 9 W a temporary worsening of electrode characteristics was noticed. After 1 day of electrode conditioning in NaCl their parameters were similar to those before irradiation. Independently, the acetone solution of compound **1** was irradiated. Immediately after the irradiation only a small amount of the *trans*–*trans* isomer was observed (TLC). On the basis of spectrum registered in d-acetone within 20 min after irradiation of compound **1** the presence of ca. 65 % of *cis*–*cis* isomer, ca. 30 % of *trans*–*cis* isomer, and ca. 5 % of *trans*–*trans* isomer was claimed.

As in all the earlier studies on the use of azobenzocrowns and bis(azobenzocrown)s as ISE ionophores, also experiments reported herein required a relatively large, i.e. minimum 3 % (w/w) content of ionophore within the membrane for successful results. In our opinion, this is a general characteristic of azobenzocrown ionophores that form sandwich-type complexes with the preferred ions as well as of other crown derivatives forming sandwich-type complexes with the main ions eg. [35]. The optimum quantity of biscrown ionophores in membranes of all type of tested electrodes is 4–5 % (w/w). For the ionophore **VI** proposed amount in membrane is 6.5 % (w/w/) [35].

*o*-NPOE was the typical plasticizer used in azobenzocrown-containing electrodes as it is considered good for sodium-selective electrodes. As shown in an example, also more lipophilic DOS may be used as the plasticizer for bis(azobenzocrown)-containing membranes. Ionophores **1**, **2**, and **3** were used in membranes of classic electrodes, membranes of miniature screen-printed planar graphite electrodes and membranes of glassy carbon-based electrodes. The latter type was studied to the greatest detail. Glassy carbon electrodes were also used for simultaneous comparative measurements using the commercial sodium ionophores: **X** (classification by Fluka)—*p*-*tert*-butylcalix[4]arene-tetraacetic acid tetraethyl ester and **VI**–bis[(12-crown-4)methyl]dodecylmethylmalonate [21].

**Fig. 6:**
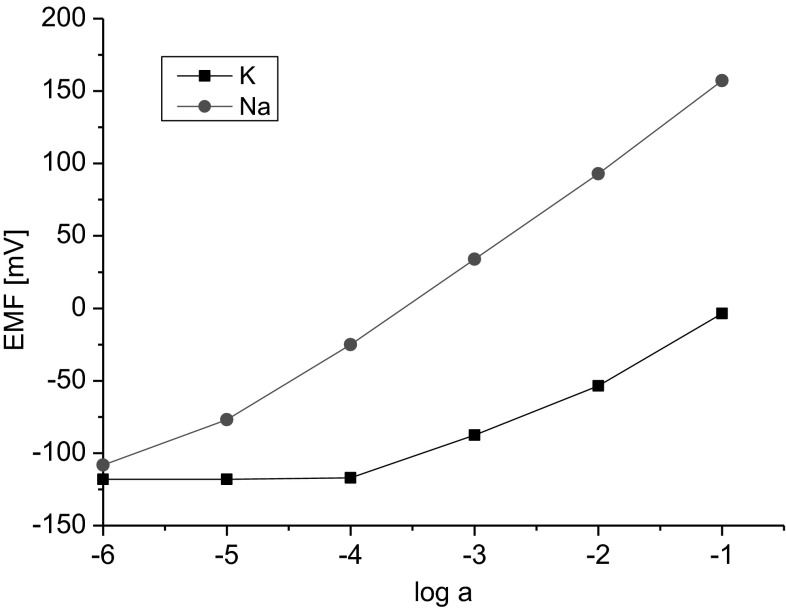
The potentiometric response of the ionophore **2**-based graphite screen-printed ISE (**s**-**p,n** type electrode) towards sodium and potassium ions

Figure 6 presents the characteristics of ionophore **2**-based graphite screen-printed electrodes, with the addition of carbon nanotubes in the membrane (**s**-**p,n** electrode) with respect to sodium and potassium ions.

Table 2 presents the selectivity coefficients determined by the SSM method (0.1 M) for classic electrodes (**c**) and two types of solid contact electrodes: graphite screen-printed (**s**-**p** and **s**-**p,n**) and glassy carbon electrodes (**gc** and **gc′**).

In Table 3 the changes in the properties of **gc′** type electrodes with ionophore **1** due to irradiation with the UV lamp with power of 9 W and changes in the properties of exposure electrodes in time are shown.

**Table 2:** Selectivity coefficients^a^ (SSM, 0.1 M) and potentiometric response characteristics of sodium selective electrodes using compounds **1**, **2, 3** and commercial sodium ionophores: **VI** and **X**

Electrode^a^	LDL [loga]	S [mV/dec]	Log*K*_*Na,K*_	Log*K*_*Na,NH4*_	Log*K*_*Na,Li*_	Log*K*_*Na,Ca*_	Log*K*_*Na,Mg*_
**1c**	−4.5	59.1	−2.5	−3.2	−3.0	−3.9	−4.5
**1** **s**-**p**	−4.5	56.7	−2.5	−3.2	−3.1	−3.9	−4.5
**1** **s**-**p,n**	−5.3	57.9	−2.5	−3.1	−3.1	−3.9	−4.4
**1gc**	−5.3	58.3	−2.5	−3.1	−2.9	−3.8	4.0
**1gc′**	−5.9	57.2	−2.5	−3.1	−2.8	−3.8	4.0
**1gc_**_**DOS**_	−4.9	60.2	−2.4	−3.2	−2.9	−3.8	−3.9
**2c**	−4.5	58.9	−2.5	−3.2	−3.1	−3.9	−4.6
**2** **s**-**p,n**	−5.3	60.0	−2.6	−3.2	−3.0	−4.1	−4.5
**2gc**	−5.3	60.9	−2.5	−2.9	−2.9	−4.0	−4.3
**3c**	−5.0	60.1	−2.2	−3.0	−2.9	−4.0	−4.7
**3** **s**-**p,n**	−5.0	60.0	−2.2	−3.0	−2.9	−4.0	−4.7
**3gc**	−5.1	59.2	−2.2	−2.9	−2.9	-4.0	−4.1
**VIgc**	−5.0	51.0	−2.0	−3.5	−3.3	−3.8	−5.0
**Xgc**	−6.0	59.4	−2.7	−3.7	−3.2	−3.8	−4.9
**0gc** ^b^	−4.0	50.0	1.8	1.2	−1.4	−1.9	−2.4

Mean values are presented for three classic electrodes, six screen-printed electrodes and three glassy carbon electrodes. Standard deviations were lower than 0.1

^a^Results for electrodes featuring *o*-NPOE plasticizer and one run for electrodes featuring DOS plasticizer-**1gc_**_**DOS**_

^b^Electrode without ionophore

***C*** classic electrode, ***s***-***p*** screen-printed graphite electrode, ***s***-***p,n*** screen-printed graphite electrode with the addition of carbon nanotubes in the membrane, ***gc, gc′*** glassy carbon electrodes)

**Table 3:** Selectivity coefficients (SSM, 0.1 M) and potentiometric response characteristics of sodium selective electrodes using compounds **1**—changes in the properties of electrodes due to membrane irradiation (UV, 9 W)

Electrode	LDL [loga]	S [mV/dec]	Log*K*_*Na,K*_	Log*K*_*Na,NH4*_	Log*K*_*Na,Li*_	Log*K*_*Na,Ca*_	Log*K*_*Na,Mg*_
**1gc′**	−5.9	57.2	−2.5	−3.1	−2.8	−3.8	4.0
**1gc′**(UV) shortly after exposure	−5.2	56.3	−2.0	−2.0	−2.0	−3.4	−3.1
**1gc′**(UV) after 1 h	−6.1	60.2	−2.2	−2.8	−2.6	−3.5	−3.6
**1gc′**(UV) after 20 h	−6.1	59.7	−2.5	−2.9	−2.7	−3.8	−3.9

**gc′**—glassy carbon electrode, *o*-NPOE plasticizer (UV)—irradiated electrode

Sodium/potassium selectivity coefficients were analyzed in particular. Electrodes with the tested biscrowns **1** and **2** were found to have better selectivity coefficients *K*_*Na,K*_ than the electrodes with the monocrown **3**. No significant difference in potentiometric responses of electrodes featuring ionophores **1** and **2** was observed; however, the best selectivity coefficient was achieved for the **s**-**p,n** electrode with compound **2** as the ionophore (log*K* = −2.6). In addition, compound **2** was obtained in higher yields than compound **1**, however so far much more research was done with compound **1**. No significant differences were also observed between the selectivities of the classic and *solid contact* electrodes; however, better (lower) limits of detection may be obtained for *solid contact* electrodes without internal electrolyte content. Particularly suitable from the standpoint of the stability of potential, detection limit and selectivity of biscrown-based electrodes was the addition of carbon nanotubes into the membrane cocktail deposited onto graphite screen-printed electrodes (**s-p,n** electrodes). The electro-conductive material was introduced directly into the membrane in a manner analogous to that proposed by Ivaska et al. [29]. In a case of glassy carbon electrodes (**gc** electrodes), the conductive PEDOT/PSS polymer blend was used as the intermediate layer between the membrane and glassy carbon. Preliminary studies, with the use of ISEs based on azobenzocrowns, on the introduction of the intermediate layer of the conductive polymer by electropolymerization were done (**gc′** electrodes). Obtained in this way electrodes revealed better LDL than electrodes **gc**. Selectivity did not change significantly. Electrodes **s**-**p,n** type and **gc** type have similar characteristics but only **gc** electrodes can be used for 3 weeks or more (in continuous conditioning in NaCl solution (10^−3^ M)) without changes of properties (see Fig. 9).

**Fig. 7:**
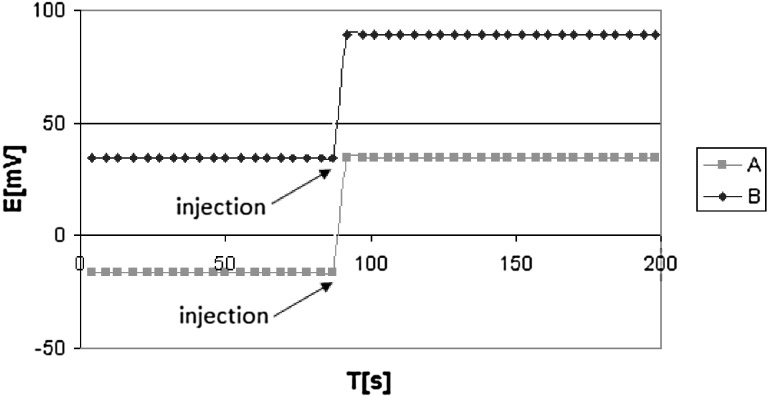
Response time of the glassy carbon electrode with a membrane featuring ionophore **1** (*o*-NPOE). (*a*) 0.9 mL of NaCl solution (0.1 M) was injected to 100 mL of NaCl solution (10^−4^ M). (*b*) 0.9 mL of NaCl solution (1 M) was injected to 100 mL of NaCl solution (10^−3^ M)

Direct measurements were used to compare the characteristics of an electrode with a membrane featuring biscrown **1** with the characteristics of electrodes with a membrane featuring sodium ionophore **VI** or sodium ionophore **X** (glassy carbon electrodes). The electrode featuring sodium ionophore **X** was found to have a slightly better Na/K selectivity as compared to the electrode with ionophore **1** (ca. 0.2 unit). A better detection limit was also observed for the electrode featuring sodium ionophore **X**. On the other hand, the electrodes with ionophore **VI** and membrane composition as proposed in the Fluka catalogue and literature [35] reveal worse properties than not only novel, reported here biscrowns but also monoazobenzocrown **3**. Electrodes featuring the tested ionophores **1**–**3** are characterized by response times not longer than 10 s (Fig. 7).

ISEs with 13-membered azobenzocrowns, particularly azobenzocrowns substituted at position *para* relative to the azo group are more pH-sensitive than ISEs featuring larger, 16-membered azobenzocrowns; however, they also meet the pH-sensitivity criterion required in medical diagnostics. Typical selectivity coefficient log*K*_*Na,H*_ for electrodes with other ester derivatives of azobenzocrowns was 1÷1.5 [2]. However, electrodes with tested biscrowns turned to be less sensitive for pH than electrodes bearing other derivatives of azobenzo-13-crown-4. Typical selectivity coefficient logK_Na,H_ for electrode with compound **1** is 0.5.

**Fig. 8:**
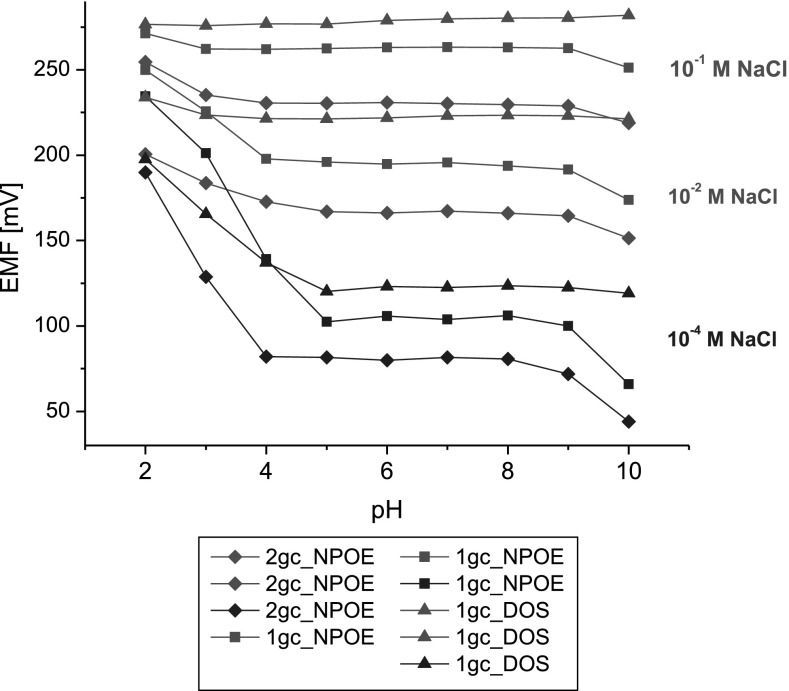
The effect of pH on the responses of electrodes in the presence of sodium at different concentrations

The effect of pH on responses of bis(azobenzocrown)-featuring membrane electrodes was also studied at a constant concentration of sodium ions (Fig. 8). For NaCl concentration of 0.1 M, constant potentials were maintained in the pH range of 3–9 for electrodes featuring *o*-NPOE (ionophores **1** and **2**) and 2–10 for the electrode featuring DOS (ionophore **1**). At NaCl concentration of 10^−4^ M, the ranges were narrowed to 4–9, 5–9, and 5–10 for ionophore **2**-*o*-NPOE, ionophore **1**-*o*-NPOE and ionophore **1**-DOS electrodes, respectively.

**Fig. 9:**
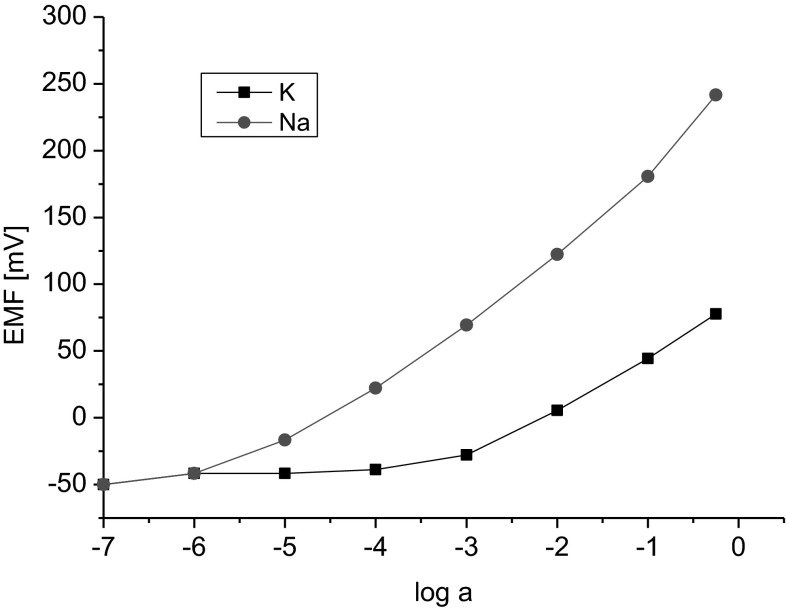
Characteristics of the response of electrode **1gc** to sodium and potassium ions after three-week conditioning in NaCl solution (10^−3^ M)

Lipophilicity of new ionophores **1**–**3** was determined as the factor influencing the electrode lifetime. Lipophilicity (TLC) of bis(azobenzocrowns) was in the range of logP_TLC_ = 12–13. Figure 9 presents the characteristics of glassy carbon electrode featuring compound **1** as a ionophore, recorded after the electrode had been conditioned for 3 weeks in NaCl solution (10^−3^ M). Of note is also the absence of the anionic effect at the activity of 1 M which is also characteristic for electrodes featuring azobenzocrown ionophores. Those electrodes can be successfully used with activities higher than 0.1 M.

In such uses of colored azobenzocrowns, their color is useful only in the monitoring of potential washout of the ionophore from the electrode membrane. In case of the tested electrodes, no washout was observed even following prolonged conditioning.

**Fig. 10:**
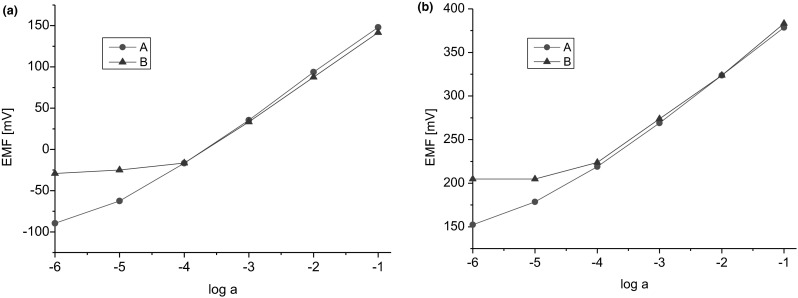
Response curves for Na^+^ obtained with ISEs based on ionophore **2. a** graphite screen printed electrode (**s-p,n**). **b** glassy carbon electrode (**gc**).* Curve A* indicates the response for Na^+^ without interfering ions.* Curve B* indicates the response for Na^+^ with interfering ions (4.2 mM K^+^, 1.1 mM Ca^2+^, 0.6 mM Mg^2+^)

Since we are interested in verification of the applicability of the synthesized ionophores in ISEs useful for clinical analysis, we evaluated the responses of the electrodes to variable concentrations of sodium ions in the presence of potassium, calcium and magnesium ions at concentrations corresponding to their respective blood plasma levels. Figure 10 presents the comparison of sodium response characteristics in the presence and in the absence of interfering ions. A near-Nernst response was recorded in the presence of interfering ions in the concentration range of above 10^−4^ M.

Glassy carbon electrodes featuring membranes with compounds **1** and **2** were used for determination of sodium levels in a real-life sample, i.e. human blood plasma. The obtained results were comparable to the result obtained in an independent measurement conducted in a clinical analytical laboratory (CAL, potentiometric method, Table 4). The most approximate result was obtained for the electrode featuring ionophore **2** and NPOE as plasticizer.

**Table 4:** Determination of blood plasma sodium levels using glassy carbon electrodes (*n* = 3 in any case) featuring bis(azobenzocrown)s as ionophores and the result of independent determination in a hospital clinical analytical laboratory (CAL)

Electrode	**1gc**_NPOE	**1gc**_DOS	**2gc**_NPOE	CAL
Sodium concentration (mmol)	129.4	129.6	129.7	130.0

## Conclusions

Three novel sodium ionophores suitable for ISEs to be used in clinical analysis were obtained, including two highly lipophilic (logP_TLC_ = 12–13) bis(azobenzocrown)s—diester derivatives of dodecylmethylmalonyl acid (compounds **1** and **2**) and monoazobenzocrown ester **3**. These ionophores proved to be very efficient in miniature *solid contact* electrodes, both graphite screen-printed and glassy carbon electrodes. Typical value of the sodium/potassium selectivity coefficient is log*K*_*Na,K*_ = −2.5 (SSM, 0.1 M, with main ion conditioning). The electrodes featuring the novel ionophores also proved to be efficient in contact with human blood plasma.

A number of ionophores for sodium cations were earlier synthesized and practically used as the Na^+^ sensing component for the ISEs. However, among commercial ionophores, only a calixarene derivative and a costly derivative of 16-crown-5 afforded Na/K selectivities that were slightly better (Sodium ionophore **X**) or better (Sodium ionophore **IV**) [21] than those of the lipophilic biscrowns **1** and **2** presented herein. Electrodes with tested biscrowns proved to be significantly better than the electrodes with structurally similar ionophore **VI**, both in terms of selectivity and the slope values of the electrode characteristics.
